# The role of HR-HPV integration in the progression of premalignant lesions into different cancer types

**DOI:** 10.1016/j.heliyon.2024.e34999

**Published:** 2024-07-22

**Authors:** Oscar Catalán-Castorena, Olga Lilia Garibay-Cerdenares, Berenice Illades-Aguiar, Hugo Alberto Rodríguez-Ruiz, Ma. Isabel Zubillaga-Guerrero, Marco Antonio Leyva-Vázquez, Sergio Encarnación-Guevara, Luz del Carmen Alarcón-Romero

**Affiliations:** a Research in Cytopathology and Histochemical Laboratory, Faculty of Chemical and Biological Sciences, Autonomous University of Guerrero, Chilpancingo, Guerrero, 39089, Mexico; b Molecular Biomedicine Laboratory, Faculty of Chemical-Biological Sciences, Autonomous University of Guerrero, Chilpancingo, Guerrero, 39089, Mexico; c CONAHCyT-Autonomous University of Guerrero, Chilpancingo, Guerrero, 39089, Mexico; d Center for Genomic Sciences, UNAM, Cuernavaca, Morelos, 62210, Mexico

**Keywords:** HR-HPV, Integration events, Cervical cancer, Head and neck cancer, Anal cancer, Penile cancer, Vaginal and vulvar cancer

## Abstract

High-risk human papillomavirus (HR-HPV) is associated with the development of different types of cancer, such as cervical, head and neck (including oral, laryngeal, and oropharyngeal), vulvar, vaginal, penile, and anal cancers. The progression of premalignant lesions to cancer depends on factors associated with the host cell and the different epithelia infected by HPV, such as basal cells of the flat epithelium and the cells of the squamocolumnar transformation zone (STZ) found in the uterine cervix and the anal canal, which is rich in heparan sulfate proteoglycans and integrin-like receptors. On the other hand, factors associated with the viral genotype, infection with multiple viruses, viral load, viral persistence, and type of integration determine the viral breakage pattern and the sites at which the virus integrates into the host cell genome (introns, exons, intergenic regions), inducing the loss of function of tumor suppressor genes and increasing oncogene expression. This review describes the role of viral integration and the molecular mechanisms induced by HR-HPV in different types of tissues. The purpose of this review is to identify the common factors associated with the role of integration events in the progression of premalignant lesions in different types of cancer.

## Highlights

• The paradigm about viral integration of HPV-HR-induced overexpression of E6 and E7 oncoproteins needs to be considered.
• There are two types of integration in which one copy or multiple tandem repeats of the viral genome are integrated.
• HPV-HR integration in cancers alters immortalization, genomic instability, cell death resistance, immune evasion, energetics, and metastasis.

## Graphical abstract

**Figure:**
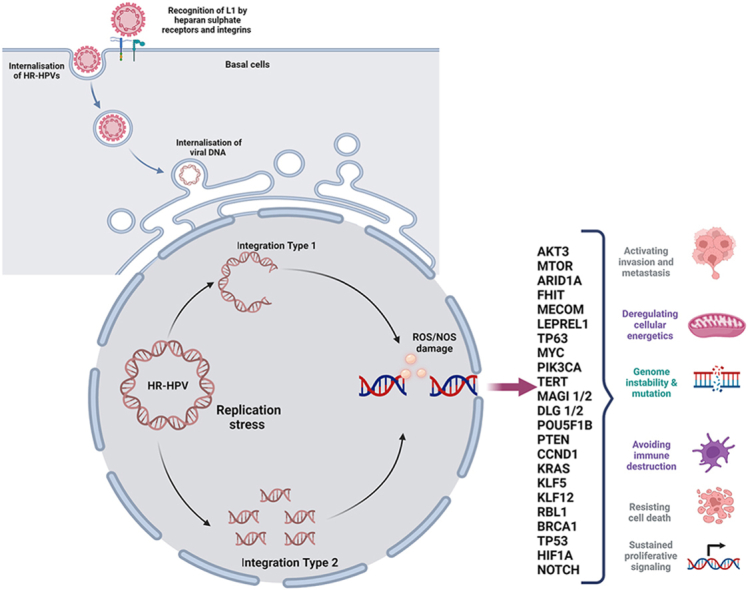

## 1 Introduction

Cancer is a multifactorial disease with multiple risk factors associated with its occurrence and progression. Oncogenic human papillomaviruses (HPVs) are among the primary etiological agents involved in the origins of different types of cancer, such as cervical cancer [1]**,** anal cancer [2], head and neck cancer [3], penile cancer [4], and vaginal and vulvar cancers [5].

Most HPV infections resolve spontaneously [6]. However, the persistence of some high-risk HPV (HR-HPV) strains promotes premalignant cervical lesions, and their chronicity induces progression to invasive cervical cancer [7]. The progression of premalignant lesions associated with papillomaviruses results from the ability of certain HPVs to integrate their genome into the host cell [8]. These integration events induce structural changes in the host cell genome that can subsequently trigger the expression of viral oncoproteins such as E6 and E7 [9], affecting the function of some tumor suppressor genes and protooncogenes that are directly correlated with the progression of cervical lesions [10,11]. Dysregulated E6 and E7 expression in host cells is a critical event in the malignant progression promoted by HPV [12]. However, there is evidence that the interaction of HPV with the host cell involves factors beyond the expression of viral oncoproteins, suggesting that the molecular events triggered by viral integration are components that determine viral oncogenesis [13].

In the present review, we analyzed the molecular mechanisms that are altered during the integration of the HR-HPV genome into the host cell genome; their effects on the progression of cancers such as cervical, head, and neck (including the oral cavity, oropharynx, and larynx), vulva and vagina, and penile and anal cancers; and how integration induces the progression of premalignant lesions to cancer in different target tissues.

## 2 Methods

A literature review of the PubMed database was performed, including information published over the last 10 years (2013–2022). This research focused on original articles evaluating HR-HPV integration loci in different cancer types using NGS, WGS, HIVID and WES. The keywords used for the search were HR-HPV, integration, next-generation sequencing, cervical cancer, head and neck cancer, anal cancer, penile cancer, and vulvar and vaginal cancer. Information on the primary genes affected by HR-HPV integration was extracted from 22 original articles. Information on these studies is presented in Table 2, which includes a list of genes deregulated due to viral integration, the frequent sites of viral genome disruption, and the region where integration occurred (introns, exons or intergenic regions). Genes deregulated in more than one cancer type are highlighted in bold. Using the UniProt platform (https://www.uniprot.org/), the codes corresponding to each gene were obtained for bioinformatic analysis. To describe the main signaling pathways associated with the genes deregulated by HR-HPV integration, pathway enrichment analysis was performed using the Gene Ontology platform (https://geneontology.org/) (Fig. 3). Finally, Reactome analysis (https://reactome.org/) was performed to locate integration-associated genes associated with cancer hallmarks (Fig. 4).

**Table 1:** HPV genotype by anatomical site and lesion type.

Anatomical site	Lesion type	HPV genotype	references
Skin	Warts Epidermodysplasia verruciformis	1, 2, 3, 4, 7, 10, 26, 27, 28, 29, 41, 48, 50, 57, 60, 63, 65,75, 76, 77, 88, 95 5, 8, 9, 12, 14, 15, 17, 19, 20, 21, 22, 23, 24, 25, 36, 37, 38, 46, 47, 49, 75, 76, 80, 92, 93, 96	[191] [190] [186] [195] [202]
Oropharynx, pharynx, and oral cavity	Head and neck cancer	16, 18	[192] [196] [201]
Oral cavity	Focal epithelial hyperplasia	13, 32	[200]
Larynx	Laryngeal papillomatosis	6, 11	[197]
Vulva and vagina	Genital warts Premalignant lesions and cancer	6, 11 16, 18	[194] [5] [193]
Uterine cervix	Cervical intraepithelial neoplasia and cancer	16, 18, 31, 33, 35, 39, 45, 51, 52, 56, 58, 66, 68, 73, 82	[21] [198] [199]
Penis	Genital warts Neoplasia and penile cancer	6,11 16,18	[189] [188]

**Table 2:** Genes deregulated by integration in different cancer types.

Cervical cancer					
Reference	Study population	Methodology	Region of the viral genome disrupted by integration	Frequent site integration	Major deregulated genes (UniProt code)
[205]	47 cervical carcinoma biopsies positive for HPV 16	APOT analysis	E1, E2 and E5	Introns, exons, and intergenic regions	**MYC (P01106)** **KLF5 (Q13887)** **KLF12 (Q9Y4X4)** HIF1A (Q16665) CASZ1 (Q86V15) GPN(Q9HCN4) MBD5 (Q9P267) ORC2 (Q13416) PARD3B (Q8TEW8) ERBB4 (Q15303) FHIT (P49789) MECOM (Q03112) ATP11B (Q9Y2G3) CLDN1 (O95832) IL8 (P10145) DUX4L2 (P0CJ85) SLC29A1 (Q99808) RUNX2 (Q13950) CREB5 (Q02930) POU5F1B (Q06416) PDK3 (Q15120)
[137]	Cervical biopsies 10 CIN I 9 CIN II 7 CIN III 89 SCC 15 adenocarcinoma	high-throughput viral integration detection (HIVID) and whole-genome sequencing (**WGS**)	E1, E2, L1, L2 and LCR	Introns, exons, flanking regions of genes	POU5F1B (Q06416) **FHIT (**P49789) **KLF12 (Q9Y4X4)** **KLF5 (Q13887)** **LRP1B (Q9NZR2)** LEPREL1 (Q8IVL5) **HMGA2 (P52926)** DLG2 (Q15700) SEMA3D (O95025) **MYC (P01106)**
[156]	72 cervical cancer biopsies with HR-HPV (16, 18, 33, 45, 51, 45, 68)	Capture-HPV-NGS	E1, E5, L1 and L2	Intergenic regions, introns, exons, and repeat regions.	**RB1 (**P06400) **KLF5 (Q13887)** **KLF12 (Q9Y4X4)** AKT3 (Q9Y243) STS (P08842) AFF3 (P51826) BCL6 (P41182) RAB11A (P62491) RAB22A (Q9UL26) **MAGI2 (Q86UL8)**
[128]	47 squamous cell carcinoma biopsies (HPV 16, 18, and 58)	HPV capture technology combined with next-generation sequencing,	E1, E2, L1, L2, E6, E7 and LCR	Introns, exons, and intergenic regions	CACNG7 (P62955) CAPG (P40121) HDAC4 (P56524) **TP63 (O88898)** CAGE1 (Q8TC20) MACROD2 (A1Z1Q3) NFIB (O00712)
[150]	Liquid-based cytology 13 ASCUS 5 CIN 1 7 CIN 2-3 7 SCC HPV 16, 18, 33, 58, 51	Miseq benchtop sequencer	E1, E2, L1 and L2	Introns and non-coding regions	**MYCN (P04198)** RRM1 (P23921) **KLF12 (Q9Y4X4)** UBXN1 (Q04323) TMEM223 (A0PJW6) ZBTB3 (Q9H5J0) TMEM179B (Q7Z7N9) UBXN1 (Q04323) UQCC3 (Q6UW78) SLC3A2 (P08195) KTN1 (Q86UP2) FBXO34 (Q9NWN3) CGRRF1 (Q99675) BMP4 (P12644) FERMT2 (Q96AC1) DDHD1 (Q8NEL9) STYX (Q8WUJ0) PSMC6 (P62333)
[117]	Cervical squamous cell carcinoma stage IIB VPH 16	Nanopore MinION Sequencer	L2, L1, E1, E2	Intergenic regions, introns, and exons	CHMP4B (Q9H444) KIF3B (O15066) ASXL1 (Q8IXJ9) ATRN (O75882) RPS6KA3 (P51812) CNKSR2 (Q8WXI2) CAGE1 (Q8TC20) **RBL1 (P28749)** **TP63 (O88898)** TRAF1 (Q13077) DCDC1 (M0R2J8) PTPRQ (Q9UMZ3) MYF6 (P23409)
[157]	Cytology samples 12 LSIL 2 HSIL 3 ASCUS HPV 39, 52,51, 51, 52 and 45	HIVID-NGS HPV	L1, L2, E7, E1, E2, E5 and E6	Intergenic regions, introns, exons, and repeat regions.	**FHIT (P49789)** CSMD1 (Q96PZ7) **LRP1B (Q9NZR2)** CSMD3 (Q7Z407) ROBO2 (Q9HCK4) SETD3 (Q86TU7) RAD51B (O15315) **MACROD2 (A1Z1Q3)**
**Head and neck cancer**					
[13]	35 head and neck tumors with HPV 16, 33 and 35	Whole-genome sequencing (**WGS**)	E1, E4 and E5	Introns, exons, the promoter region	RAD51B ((O15315) ETS2 (P15036) PDL1 (Q9NZQ7) NR4A2 (P43354) **TP63 (O88898)** **KLF5 (Q13887)** BARX2 (Q9UMQ3)
[104]	7 head and neck cancer cell lines (HPV 16 and 18)	Whole-genome sequencing (**WGS**)	*Does not specify the region of the viral genome	Exons, introns, and intergenic regions	DIAPH2 (O60879) **TP63 (O88898)** FOXE1 (O00358) PIM1 (P11309) SLC47A2 (Q86VL8) ACTBL2 (Q562R1) PTEN (P60484) **PIK3CA (P42336)** NOTCH1 (P46531) SYNE1 (Q8NF91) SYNE2 (Q8WXH0) CASP8 (Q14790)
[143]	7 head and neck cancer cell lines (HPV 16 and 18)	**DIPS-PCR**	E1, E2, L1 and LCR	Intergenic regions, Introns, Exons, and promoter region	**TP63 (O88898)** JAK1 (P23458) TERT (O14746) ATR (Q13535) ETV6 (*P*4_1_2_1_2) PGR (P06401) PTPRN2 (Q92932) TMEM237 (Q96Q45)
[151]	149 oral squamous cell carcinomas positive for HPV 16, 18, 33, 35, 59, and 69.	Whole genome sequencing (**WGS**) and Whole exome sequencing (**WES**).	* Does not specify the region of the viral genome	Exons, introns, and intergenic regions	**PIK3CA (P42336)** ZNF750 (Q32MQ0) FGFR3 (P22607) CASZ1 (Q86V15) PTEN (P60484) CYLD (Q9NQC7) DDX3X (O00571) ZNF750 (Q32MQ0) EP300 (Q09472) CASZ1 (Q86V15) **RBL1 (P28749)** IFNGR1 (P15260) NFKBIA (P25963) NSD1 (Q96L73) ASAP1 (Q9ULH1) BBX (Q8WY36) SLTM (Q9NWH9) STAT1 (P42224) ZNF750 (Q32MQ0) FBXW7 (Q969H0) NSD1 (Q96L73) TGFBR2 (P37173)
[161]	36 head and neck tumors positive HPV 16 and 18	**DIPS-PCR**	E1, L1, E2 y L2	Intergenic regions, introns, and exons	PTPRN2 (Q92932) SCN1B (Q07699) YIPF1 (Q9Y548) SGCZ (Q96LD1) DNAI1 (Q9UI46) NPAS3 (Q8IXF0) UTP18 (Q9Y5J1) RLN1 (P04808) KIF21B (O75037)
**Anal cancer**					
[159]	61 biopsies of squamous cell anal carcinoma (VPH-16 y 18	Hybrid-capture-based next-generation sequencing of exons	*It does not specify the region of the viral genome*	Introns and exons	**PIK3CA (P42336)** MLL2 (O14686) **PTEN (**P60484) SOX2 (P48431) FBXW7 (Q969H0) **TP53 (P04637)** **MYC (P01106)** RICTOR (Q6R327) FGF10 (O15520) FGF3 (P11487) CCND1 (P24385) FBXW7 (Q969H0) STK11 (Q15831) **AKT1 (P31749)** **AKT2 (P31751)** **AKT3 (Q9Y243)**
[160]	40 anal squamous cell carcinoma biopsies HPV 16-positive	Whole exome sequencing (**WES**)	*It does not specify the region of the viral genome*	Intergenic regions, Introns, Exons, and promoter region	FBXW7 (Q969H0) **PIK3CA (P42336)** **TP63 (O88898)** **EP300 (**Q09472)
[204]	20 cases of anal squamous cell carcinoma positive for HPV 16	Whole exome sequencing (**WES**)	*It does not specify the region of the viral genome*	Introns, exons, and intergenic regions	**PIK3CA (P42336)** **MYC (P01106)** **TERT (**O14746) DDR2 (Q16832) **CCND1 (**P24385) MDM2 (Q00987) **AKT2 (P31751)** FAT1 (Q14517) FBXW7 (Q969H0) TRIP12 (Q14669) CYLD (Q9NQC7) **PTEN (**P60484) BAP1 (Q92560) **FHIT (P49789)** SETD2 (Q9BYW2) MLH1 (P40692) TGFBR2 (P37173) CTNNB1 (P35222) PBRM1 (Q86U86) MCM4 (P33991) NOTCH1 (P46531) DDX5 (P17844)
[142]	72 anal squamous cell carcinoma tumors HPV 16, 18 y 6	whole-exome sequence (WES)	E4, L1, L2 and E2	Introns, exons, promoter, and intergenic regions	BRCA2 (P51587) FOXO1 (Q12778) RB1 (P06400) ATR (Q13535) FANCD2 (Q9BXW9) FHIT (**P49789**) MLH1 (P40692) SETD2 (Q9BYW2) MSH3 (P20585) PARP3 (Q9Y6F1) RAD18 (Q9NS91) RAD50 (Q92878) XPC (Q01831) **CCND1 (**P24385) MYC (**P01106)** NOTCH1 (P46531) TERT (O14746) PIK3CA (**P42336)**
[152]	4 anal cancer cell lines HPV 16 positive	Whole-exome sequencing (WES)	*It does not specify the region of the viral genome*	Intergenic regions, Introns, Exons, and promoter region	**PIK3CA (P42336)** PIK3CB (P42338) **TP63 (O88898)** SOX2 (P48431) FGF10 (O15520) **TERT (**O14746) **RICTOR (**Q6R327) STK3 (Q13188) **MYC (P01106)** ABL1 (P00519) **NOTCH1 (**P46531) **CCND1 (**P24385) BCL2L1 (Q07817) TRIP12 (Q14669) COL6A3 (P12111 IDH1 (O75874) ERBB4 (Q15303) PAX3 (P23760) FGFR1 (P11362) MGMT (P16455) EED (O75530) ATM (Q13315) NTRK3 (Q16288) SMAD4 (Q13485) ARID3A (Q99856) SERPINB11 (Q96P15) STK11 (Q15831)
**Penile cancer**					
[163]	HPV-positive squamous cell penile carcinoma biopsies: 16, 53, (16/40), (16/62), and (18/40)	Array comparative genomic hybridization (aCGH) combined with HPV infection status	*It does not specify the region of the viral genome*	Intergenic regions, Introns, Exons, and promoter region	DLC1 (Q96QB1) PPARG (P37231) LAMP3 (Q9UQV4) **MYC (P01106)**
[153]	HPV-positive squamous cell penile carcinoma biopsies: 16, 51, 33, 56	HIVID (high-throughput Viral Integration Detection)	L1, L2, E1, E2 and LCR	intragenic regions, introns, promoter región, and 5′ untranslated, 3′ untranslated regions	**KLF5 (Q13887)** **LRP1B (Q9NZR2)** **KLF12 (Q9Y4X4)** CADM2 (Q8N3J6) CEP19 (Q96LK0) CSMD1 (Q96PZ7) NRROS (Q86YC3) CMC2 (Q9NRP2)
**Vulvar cancer**					
[166]	43 biopsies of vulvar squamous cell carcinoma positive for HPV 16, 18, 31 and 33	Next-generation sequencing (NGS)	* Does not specify the region of the viral genome	* Somatic mutations were evaluated	**TP53 (O88898)** **PIK3CA (P42336)** **CDKN2A (Q8N726)** HRAS (P01112) **PTEN (**P60484) FGFR3 (P22607) KIT (P10721) CTNNB1 (P35222) APC (P25054) **KRAS (P01116)** ERBB4 (Q15303) SMARCB1 (Q12824) FLT3 (P36888)
[167]	6 biopsies of vulvar squamous cell carcinoma positive for HPV 16, 52, 58	NGS-based whole-exome sequencing(WES)	*It does not specify the region of the viral genome*	Intergenic regions, Introns, Exons, and promoter region	**PIK3CA (P42336)** **BRCA2 (P51587)** FBXW7 (Q969H0) ERC1 (Q8IUD2) CDK12 (**Q9NYV4)** NRG1 (Q02297) **TP53 (O88898)** FAT1 (Q14517) CASP8 (Q14790) **SMAD2 (Q15796)**
[154]	102 biopsies of vulvar squamous cell carcinoma positive for HPV 16, 18, 31, 33, 58 and 67	De novo assembly of nonhuman sequencing reads and nucleotide Basic Local Alignment Search Tool (BLASTn)	*It does not specify the region of the viral genome*	Intergenic regions, Introns, Exons, and promoter region	**PIK3CA (P42336)** **PTEN (**P60484) EP300 (Q09472) STK11 (Q15831) FBXW7 (Q969H0) SOX2 (P48431) **MTOR (P42345)** BAP1 (Q92560) PBRM1 (Q86U86) KMT2C (Q8NEZ4) **ARID1A (O14497)** **RB1 (**P06400) **CDK12 (Q9NYV4)** FGFR3 (P22607)

## 3 Epidemiology

Approximately 630 000 HPV-related neoplasms are diagnosed worldwide each year, including 530 000 associated with cervical cancer, 35 000 with anal cancer, 29 000 with oropharyngeal cancer, 4400 with oral cavity cancer, and 3800 with laryngeal cancer, 13 000 with penile cancer, 12 000 with vaginal cancer, and 8500 with vulvar cancer [14,15]. HPV genotype-attributable lesions and cancer vary by anatomical site (Table 1). The carcinogenic effects of HPV are not limited to the uterine cervix; it is estimated that 88 % of anal, 70 % of vaginal, 50.8 % of penile, and 50 % of anal cancers are caused by HPV [16]. In addition, 43 % of vulvar and 13–56 % of oropharyngeal cancers are attributable to HPV 16 and 18 [5].

The International Agency for Research on Cancer (IARC, https://www.iarc.who. int/) considers HPV types 16, 18, 31, 33, 35, 39, 45, 51, 52, 56, 58, 59, 66, 68, 73 and 82 to be Group 1 carcinogens in humans, and these types are therefore considered to be HR-HPV. HPV 16 and 18 are associated with 75 % of invasive squamous cell carcinoma (ISCC) cases in patients with cervical cancer. In comparison, the incidence of HPV-related adenocarcinoma is 21 %, with HPV 18 being the leading etiological agent, and 4 % of cases are cervical adenosquamous carcinoma [17].

Moreover, HPV 16 is the most prevalent type of infection worldwide; HPV 33 and 31 are the most prevalent in Europe and the USA. In Africa, the most common HPV types are HPV 35 and 45 [18]. In Asia, most infections are associated with HPV 52 and 58 [19]. In Mexico, the most prevalent viral types are HPV 16, 18, 58, 31, and 45 [20,21].

## 4 Structural features of HPV

HPV is part of the *Papillomaviridae* family, which is classified into four genera (*Alpha, Beta, Gamma, Nu,* and *Mu*) and comprises various viruses that infect humans and animals [22]. The *alpha papillomavirus* genus includes genotypes associated with cancer [23]. *Alpha papillomaviruses* are nonenveloped DNA viruses that are approximately 55 nm in size; their capsid is icosahedral and composed of 72 capsomers with a circular, double-stranded genome of approximately 8000 base pairs [24,25]. The HPV genome comprises six to eight open reading frames (ORFs) that are expressed as polycistronic transcripts [26]. The genome is organized into an early region (E) consisting of the early genes E1, E2, E4, E5, E6, and E7 and a late region (L) containing the genes coding for the major (L1) and minor (L2) capsid proteins [27] and a noncoding sequence known as the long control region (LCR)**,** which contains the origin of replication and binding sites for transcription factors such as SP1 and SP2 and the promoter regions p97 (for early genes) and p670 (for late genes) that contribute to regulating viral replication and transcription [28,29].

## 5 The viral cycle

### 5.1 Target cells for HPV infection

The first mechanism that ensures viral entry into host cells is infection of the basal stratum epithelium, where symmetric cell division occurs to give rise to more basal cells. Furthermore, asymmetric division occurs, where basal cells differentiate, guaranteeing constitutive replication and the release of virions during epithelial renewal [30].

Productive HR-HPV infection begins in the uterine cervix, specifically in the squamocolumnar junction (SCJ) or squamocolumnar transformation zone (STZ), including the ectocervix and endocervix [31]. Initially, basal cells of the nonkeratinized squamous epithelium were considered the only cells targeted by HR-HPVs via microlesion exposure [32,33]. However, during reproduction, cellular re-epithelialization occurs through endocervical squamous metaplasia in response to estrogenic hormonal stimuli, and changes in the pH of the cervical mucosal frequently occur [34]; in both processes, the cells of the columnar endocervical epithelium are susceptible to infection by sexually transmitted pathogens [35,36]. The STZ also comprises a specialized type of reserve stem cell and other cuboidal-like cells that present molecular and phenotypic hallmarks of cervical intraepithelial neoplasia grade 2, 3 or higher (CIN 2–3+) and cervical cancer cells, suggesting that cuboidal cells are also involved in the development of invasive cervical carcinoma [37].

The morphological structure of the anal canal is similar to that of the cervical epithelium and consists of columnar cells (anal glands), squamous epithelia, and a transition zone containing cuboidal cells [38]. Anal STZ cells are also susceptible to HPV infection [36]; in this metaplastic epithelium, the decreased expression of keratin intermediate filaments and cell envelope components, such as involucrin and loricrin [39], reduces resistance to mechanical stress generated by cell rupture and cell death [40,41], resulting in increased vulnerability to trauma that favors the internalization of HPV in the cells of the anal STZ [35].

Head and neck cancer comprises tumors originating in the oral cavity, oropharynx, larynx, hypopharynx, and nasosinusal tract [42]. Although head and neck cancer is caused by multiple factors, such as alcohol consumption, smoking, inherited chromosomal instability, Fanconi's anemia, and Bloom's syndrome [43,44], an increase in cases associated with HPV infection has been reported [45]. HPV prefers cells in the tonsillar crypts of the palatine and lingual tonsils [46]. The mechanism of infection in tonsils is different from that in the uterine cervix given that the tonsillar surface is lined with nonkeratinizing squamous epithelium, whereas the interior of the crypts is lined with reticulated squamous epithelium [47]. This reticulate squamous epithelium has an intermediate desmosomal network that provides a scaffold-like structure linking the individual cells together, making the layer permeable and porous [48] and allowing HPV to migrate into the basal layer of the reticular epithelium [[48], [49], [50]].

The basal epithelial layer of the oropharyngeal mucosa harbors stem cells responsible for epithelial renewal [51,52], as the basal cells of the ectocervix, basal cells, and epithelial stem cells of the oropharynx express α6 integrin-like receptors on their surface, which are recognized by viral capsid proteins (L1 and L2) and therefore favor their internalization [53].

The vulvar epithelium consists of keratinized squamous epithelium, a modified mucosa with sebaceous glands, and glycogen-producing mucosa [54]. The squamous mucosa lining the vagina consists of a flat nonkeratinized epithelium similar to the uterine cervix, and it comprises a deep basal epithelium stratum, parabasal or intermediate, and superficial cells [55]. The entry of HPV into these epithelia occurs through skin abrasions, enabling the viral particles to contact the reserve stem cells of the basal stratum. Once HPV enters a basal cell, the infection may develop into a latent or nonproductive infection in which the viral DNA persists during basal cell replication without apparent morphological alterations [56]. During productive infection, viral DNA replication in the squamous epithelium's intermediate and superficial cell layers proceeds independently of host chromosomal DNA synthesis [57], producing virions that generate morphological changes in surface cells known as koilocytes [58].

Only 20 % of penile carcinomas are caused by HPV 16, which suggests that the structure of the tissue is not as permissive to infection and malignant transformation [59,60]. Because most penile intraepithelial neoplasms are associated with nonviral factors, such as chronic inflammation, lichens, and phimosis**,** information on the life cycle of HPV in the penile epithelium is limited [61]. Penile HPV infection initiates in squamous epithelial cells, with a predominance of keratinocyte stem cells, which allows for the maintenance of viral replication via HPV entry through microlesions or minor wounds in the basal layer of the penile epithelium, and the receptors responsible for HPV entry are integrin α6 and heparan sulfate (glycosaminoglycans) [62].

A high frequency of HPV infections has been observed in the penile epithelium without apparent morphological alterations [63] because its keratinized epithelium does not induce an immune response as effectively as the mucosal epithelium does [64], with glans cells being the major target cells of HPV infection, accounting for 48 % of cases, followed by the foreskin (21 %), glans and foreskin (9 %), coronal sulcus (6 %) and, less frequently, the penile shaft (<2 %) [65]. Squamous cell carcinoma of the penis (SCCP) is the most common histological penile cancer type and is subdivided into basaloid, condylomatous, keratinizing, and warty subtypes [66]. The basaloid and condylomatous subtypes of SCCP have frequencies of 80 % and 100 %, respectively, of HPV DNA. The keratinizing and warty cancer subtypes are not strongly associated with HPV infection, with percentages of HPV DNA of 34.9 % and 33.3 %, respectively [67,68].

**Fig. 1:**
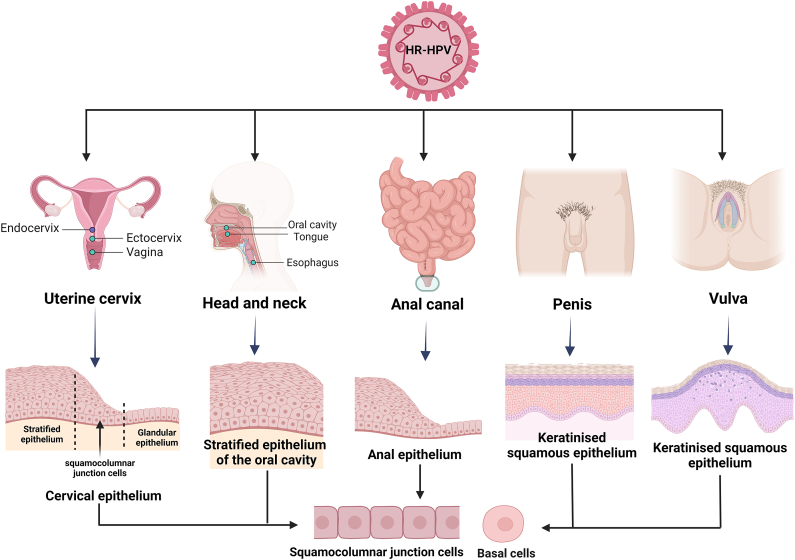
**HR-HPV targets cells from different anatomical sites.** The tissue types have typical basal or cuboidal cells in constant proliferation, which the virus uses for its proliferation and integration into the host cell genome [5,159,[186], [187], [188], [189], [190], [191], [192], [193], [194], [195], [196], [197], [198], [199], [200], [201], [202], [203], [204], [205]].

Although the frequency of HPV infection varies across different epithelia, basal cells of the flat epithelium and SZT cells (cuboidal, reserve stem cells, and metaplasia cells), which are rich in heparan sulfate proteoglycans and integrin-like receptors (α6, β4, and β1), are characterized as the primary cells targeted for viral internalization and replication (Fig. 1).

### 5.2 Internalization

**Fig. 2:**
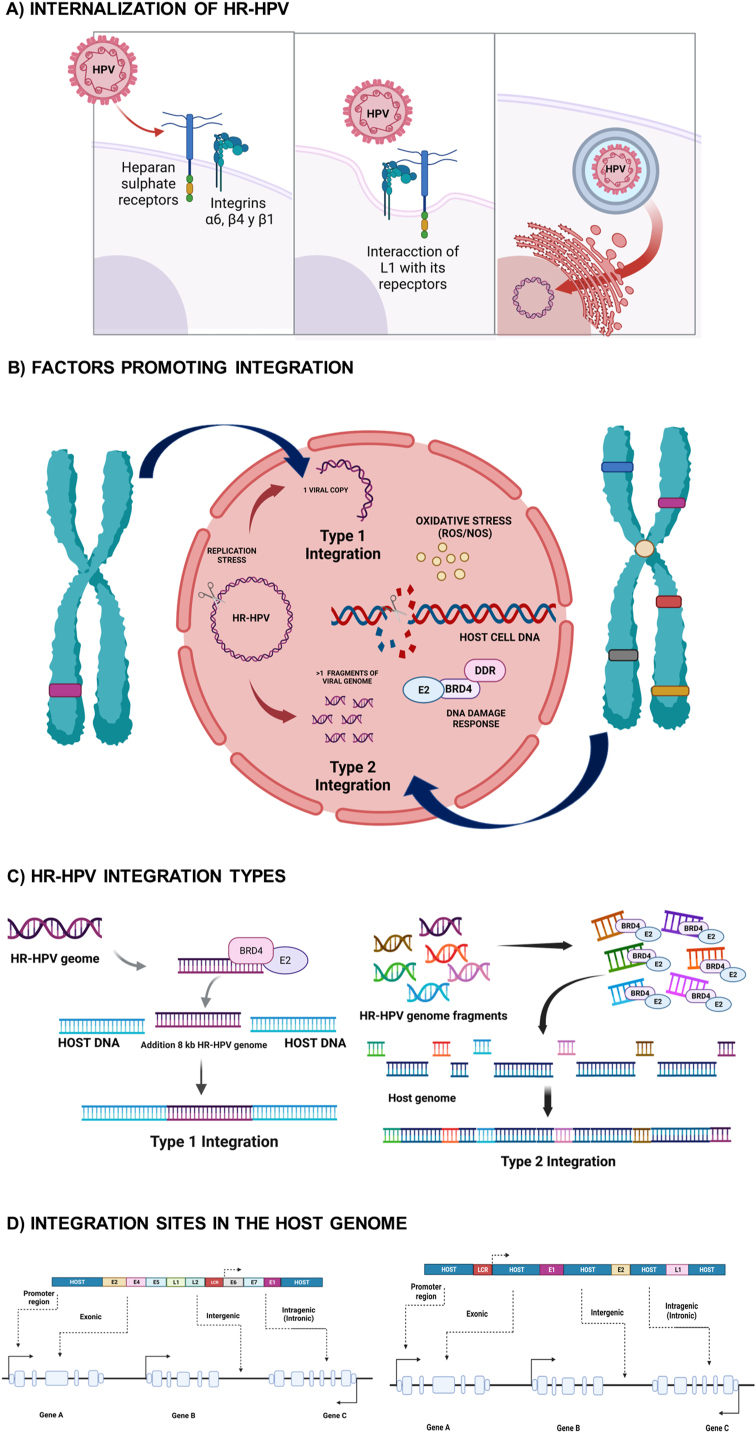
**Infection of HR-HPV in target cells in different tissues.** The infection starts with the A) internalization of the virus, which implies the recognition of other molecules of the host cell membrane that favor its endocytosis; once internalized, different factors are produced, such as replication stress that promotes the rupture of the viral and host cell genomes, tending C) the different types of integration in D) the different sites of the host cell genome [5,159,[186], [187], [188], [189], [190], [191], [192], [193], [194], [195], [196], [197], [198], [199], [200], [201], [202], [203], [204], [205]].

The internalization of HR-HPVs into basal epithelial cells begins with the interaction of the viral L1 protein with heparan sulfate proteoglycans and the α6, β4, and β1 integrins of the cells [53]. Cyclophilin B (peptidyl-prolyl cis/trans isomerase) induces a conformational change in the viral capsid [69], exposing the amino-terminal end of L2, which makes it susceptible to cleavage by an extracellular convertase (furin) [70,71]; the cleavage of L2 by furin is a prerequisite for the internalization of the virus into the host cell via endocytosis [72,73]. Once internalized, L1 is degraded, and the viral genome migrates from the trans-Golgi network into the nucleus during cell cycle progression [28,74]. Once inside the nucleus, the viral genome associates with the ND-10 complex of nuclear proteins involved in transcriptional regulation, promoting its transcription [75] (Fig. 2-A).

**Fig. 3:**
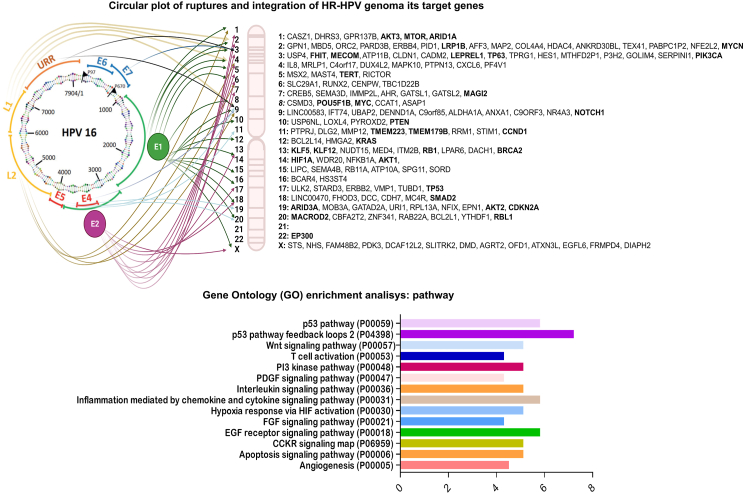
**Molecular and functional effects of integration.** The circular plot shows the frequent sites of viral genome disruption and the regular sites of viral genome insertion into host cell chromosomes and genes (genes mostly affected by integration into more than one type of cancer are highlighted in bold). Analysis of the ontology of genes involved in integration shows the main signaling pathways affected by type 2 integration, in which the effect on P53 signaling is mainly observed.

**Fig. 4:**
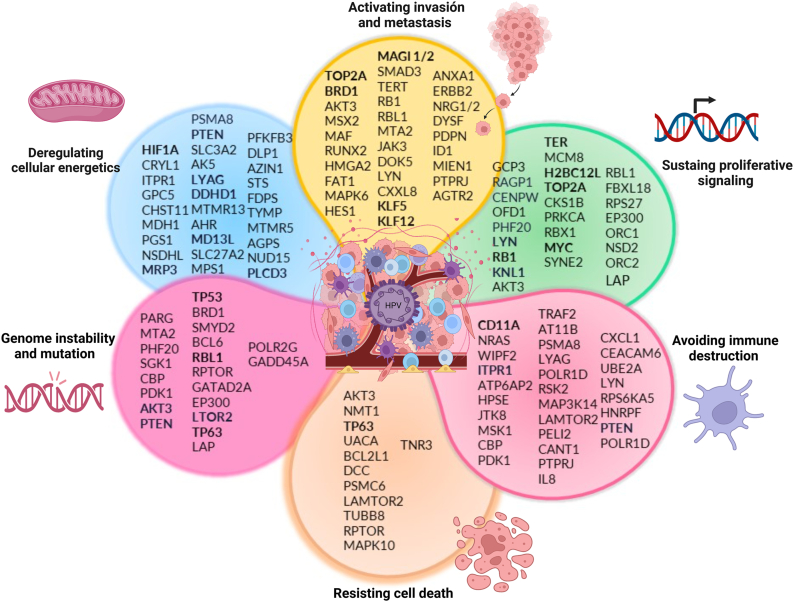
**Effect of HR-HPV integration on cancer hallmarks.** Integration of the oncogenic virus has direct effects on apoptosis, immune response evasion, metabolic plasticity, clonal expansion, cell proliferation, invasion, and metastasis, hallmarks of cancer associated with tumor progression.

The first viral mRNAs detected in infected cells encode the E1 and E2 proteins, which initiate viral replication, increasing the viral load to approximately 100 copies per cell [76]. The infection is considered nonproductive, as no new virions have been produced at this stage [77]. In infected basal cells, the viral and host DNA are simultaneously replicated and evenly distributed among the cells [75]. The last stage of the viral cycle involves the formation of icosahedral particles [78], and the L1 and L2 proteins spontaneously assemble to form a capsid containing a copy of the viral genome [79]. Given that HPV is a nonlytic virus, it takes advantage of the shedding of the upper layer of the squamous epithelium as a mechanism of release into the extracellular environment and eventually reinfects other epithelial cells [80]. Although there is a wide variety of HPV genotypes, the internalization mechanism is the same.

### 5.3 Viral protein expression

Viral proteins in HR-HPV-infected cells interact with other proteins, inducing various molecular mechanisms [81]. E1 and E2 encode proteins responsible for extrachromosomal DNA replication and viral cycle termination [82]. E1 acts as a helicase that initiates and elongates viral DNA. E2 is a DNA-binding protein that binds and recruits E1 at the origin of replication in the viral genome [83]. Nevertheless, it can also act as a transcriptional activator or repressor of viral genes [84]. The E1 gene encodes two proteins, one of which inhibits transcription of the early region of the genome, whereas E2 enhances the transcription of early genes [85]. In the basal cells of the infected epithelium, the E2 protein is fused with the open reading frame of E8; this fusion protein product represses viral replication and transcription, thus maintaining HPV in a latent state [86]. The E4 protein is expressed from a spliced mRNA (E1∧E4) found in cells where viral DNA replication occurs (productive cycle) [87]. The expression of the E1∧E4 protein of HR-HPVs triggers degradation of the network of intermediate filaments and cytokeratins, allowing the release of new viral particles and the loss of cell differentiation, giving rise to koilocytes [88]. Koilocytes are mature squamous cells with perinuclear halos; these cells have large nuclei (karyomegaly) and exhibit binucleation and hyperchromasia [89,90].

The oncogenic potential of HR-HPVs is due to two functional proteins, E6/E7**,** which can immortalize and transform infected cells; the E6 oncoprotein of HR-HPVs is found in the nucleus and cytoplasm [91]. E6 is a 151 amino acid protein that harbors an LXLL motif at its amino terminus. This region is required for the interaction of E6 with E6AP. This protein complex targets many cell proteins, the classical example of which is the tumor suppressor protein P53 [92]. The E6 protein of LR-HPVs can also bind to E6-AP and form complexes with p53 but does not lead to p53 degradation [93,94]. Another E6 motif found in the carboxyl-terminal region is the S/TXV motif (protein recognition motif known as PDZ) [[95], [96], [97]]. The HR-HPV E6 protein can bind to several host cell proteins, interfering with transcription, chromatin remodeling, cytokine signaling, protein degradation, cell polarity, apoptosis, and cellular processes that result in genomic instability [11].

The E7 protein consists of 98 amino acids separated into three conserved domains: CR1, CR2, and CR3. CR2 includes a conserved LXCXE motif that mediates high-affinity binding to pRB [98,99]. The CR3 region contains two CXXC motifs separated by 29 or 30 amino acids, forming a zinc-binding domain. This region is crucial for interactions with cellular proteins, such as pRB, p21, OCT4, p27, TBP, and E7-E2F [100]. E7 of HR-HPV induces cell proliferation by disrupting p21 and p27 cyclin-dependent kinase (CDK) inhibitor activity, activating CDKs, and destabilizing the tumor suppressor protein Rb [101]. Rb family members (pRb, p107, and p130) are proteins that regulate cell cycle progression by regulating the transcription factor E2F. Therefore, the binding of E7 to hypophosphorylated Rb induces the release of the E2F factor necessary for the transcription of genes involved in cell cycle progression [102,103].

## 6 Viral integration

Among the factors associated with HR-HPV oncogenicity, viral integration into the host cell genome can induce mutations and modify the pattern of gene expression, consequently affecting the expression and integrity of proteins involved in cancer progression [104,105]. However, integration is not part of the normal life cycle of HPV in contrast to retroviruses, which encode integrase-type enzymes that promote the insertion of their genome into the host cell genome [106].

### 6.1 Factors that promote integration

The integration rate of HR-HPVs varies according to the type of epithelium infected and the viral genotype. In cervical squamous cell cancer, the degree of viral genome integration is 92 % for HPV 16 and 100 % for HPV 18 [107], and the viral genome integration is observed in 53.3 % of cervical adenocarcinoma cases. In head and neck cancer, integration occurs in 71.2 % of cases (13), and HPV16 genome integration occurs in 71 % of anal carcinoma cases [108].

The integration provides the infected cell with a selective growth advantage given the genomic instability caused by viral E6/E7 gene expression [109]. According to the Darwinian model of neoplastic evolution, clones of mutant cells that acquire a dominant competitive advantage are positively selected for persistence [110]. In stratified epithelia, clonal expansion is associated with p53 and pRb inhibition [111]. Increased E6 levels reduce the proportion of differentiated cells within the infected basal cell population, allowing their clonal expansion and persistence with the transformation of large areas of the epithelium. This phenomenon results in neoplastic progression because of viral genome integration. During HPV infection and internalization in any epithelium, the viral genome can be in an episomal, integrated, or mixed (episomal and integrated) state [112]. Although HR-HPV is integrated into high-grade lesions and cancer**,** it has been reported that HPV 16 can also integrate into patients without an apparent lesion or at the onset of active viral infection [113].

HR-HPV genome integration may occur randomly at the onset of infection (in premalignant lesions); however, in conditions with persistent viral infection, viral genome integration is observed at recurrent loci [114,115], as it appears to provide clonal selection advantages that contribute to carcinogenesis because genes at these "fragile sites" are continuously expressed during transcription and DNA repair [116,117].

For efficient viral genome integration to occur, damage to the DNA of both the host cell and the virus must occur, which can be caused by exposure to reactive oxygen and nitrogen species (ROS/NOS) generated by E1-mediated viral replication [118,119]. Damage to host cell DNA is repaired by activating the DNA damage response (DDR) [120]. However, E6 promotes nonrepair damage through the degradation of p53, which senses or recognizes cleavage sites [121]. The genome integration event requires proximity between the damaged DNA of both the host cell and the virus. During the virus life cycle, its genome binds to host cell DNA chromatin through the formation of the E2 complex with bromodomain protein 4 (BRD4), a chromatin reader protein that recognizes and binds to acetylated histones through cell division, regulating transcription at common fragile sites [122]. BRD4 activates viral transcription at this integration site and promotes E6/E7 expression, leading to the formation of an oncogenesis-promoting element [123] (Fig. 2-B).

Viral genome integration depends on the type of HPV and the epithelium infected, with HPV 16 being more likely to integrate into the host DNA. This genotype is more frequently found in premalignant lesions and cervical cancer than in head and neck cancer and penile cancer [124], with a frequency of 74.1 %, whereas HPV 18 and HPV 45 exhibit integration frequencies of 15 % and 3.5 %, respectively [125].

### 6.2 Integration types

Integration randomly occurs in introns, exons, intergenic regions, or even in several regions of the human genome (116) associated with fragile, DNase-hypersensitive, and transcriptionally active chromosomal sites [126,127]. HR-HPVs can be integrated into genomic regions enriched with genes, CpG islands, and open chromatin [125]. HPV genome breakage sites can occur in any region, with the most frequent being the E2, E4, and E7 regions, followed by the E1, L2, and E6 regions, whereas the least frequent regions include the long control region (LCR) and E5 and L1 regions [128].

Different molecular processes associated with integration have been described; however, HR-HPV integration is classified into two types. In type 1 integration, one copy of the viral genome is integrated. In type 2 integration, multiple tandem repeats of the viral genome are found, in some cases with intermediate host flanking genomic sequences [129] (Fig. 2-C).

In general, integration promotes E6/E7 oncogene overexpression by cleaving the E2 transcriptional repressor from the p97 promoter region [130]. Nevertheless, in type 1 integration, low E6/E7 viral oncogene expression occurs because the whole genome is integrated. Its transcriptional repressor, the E2 gene, is silenced by DNA methylation [131]. However, hypermethylation of E2 binding sites in the p97 promoter region blocks E2 repression of E6/E7 and favors the transcriptional activation of oncogenes [132].

Type 2 integration is the most common method, where transcriptionally active fragments of random portions of the viral genome are generated when the promoter region and the sequence coding for E6/E7 are present, ensuring their constant expression while sustaining the proliferative potential and survival of the cells [133]. However, recently, nanopore technology analysis performed in cervical cancer (stage I-II) suggested four types of HPV 16 integration: type A, characterized by the insertion of a truncated viral genome harboring the E6/E7 coding regions; type B, characterized by the insertion of a truncated viral genome lacking the E6/E7 coding regions; type C, characterized by the insertion of the entire viral genome; and type D, the integration pattern of which involves a combination of types A, B and C [134]. However, this type needs to be analyzed in other cancer types associated with HR-HPVs.

E6/E7 transcription during type 2 integration is promoted by the binding of the viral genome with BRD4 to integration sites when multiple tandem repeats of the viral genome are present [123]. Disruption of E1 during the integration process affects the repressor function of E2. Moreover, E1 causes DNA damage that leads to genomic instability by inducing excessive amplification of the integration region [133].

Although the molecular basis of integration is associated with the initial overexpression of E6/E7 oncoproteins in HR-HPVs, viral integration occurs in a random pattern; depending on the type of integration, the type of viral breakage pattern and the sites at which it integrates into the host cell genome, integration can promote events that may favor the virus adapting to the microenvironmental changes that occur during cancer progression [135].

Homology is a determining factor for viral DNA integration into cellular DNA, with at least 80 % of gene regions including up to 34 nucleotides showing high E5 and L2 homology [136]. The analysis of genomic sequences at the integration points between cells and HPV DNA has allowed the proposal of a new model of viral integration [137], in which breaks or replication loops generated by genomic instability could activate microhomology-mediated DNA repair pathways such as fork stalling, template switching (FoSTeS) [138] and microhomology-mediated break-induced replication (MMBIR) [139]. HPVs can hijack these pathways to promote their fusion with the broken host genome and complete the integration process [137].

Once the HPV genome has been integrated, alterations or even complete deletions of the E1 and E2 genes and their subsequent functional inactivation have been observed [140]. The HPV-specific helicase essential for initiating viral DNA replication is encoded by the E1 gene [83], whereas the E2 gene encodes a regulatory protein that regulates transcription, replication initiation, and episomal maintenance of viral DNA [84]. Loss of E1/E2 gene expression overrides E2-mediated repression of E6/E7 transcription of integrated HPV DNA, increasing the efficiency of HPV-induced immortalization [141].

In cervical cancer, multiple viral genome break sites have been reported, including 18 break sites for L2, 12 break sites for L1, 11 for E1, and 7 for E2 [117]. In anal carcinomas, the type 2 integration is the most common type of integration for HPV 16 and 6, and the most frequent cleavage sites of the viral genome are located in E4, L1, L2, and E2 [142] (Fig. 2-D). The most frequent type of viral genome integration in head and neck cancer is type 2, which occurs through disruption at E2 and E1 [143].

The alterations in the host genome caused by the insertion of the viral genome can result in the loss of function of specific genes, inducing the amplification and increased expression of other genes and even inter- and intrachromosomal rearrangements, leading to mutations [144].

In recent years, *de novo* methylation of the viral genome has been described as a mechanism to prevent replication and to silence regions where the HR-HPV genome has been integrated [145]. Methylation has been reported to occur mainly in the L1 and L2 regions and CSF, and the methylation status increases with the progression of premalignant lesions to cancer [146]. In type 1 integration, the viral genome is hypomethylated; in type 2 integration, it is hypermethylated in the CSF region [147]**.** Therefore, when a single copy of the viral genome is integrated, it is transcriptionally active; however, when multiple copies are integrated, it is epigenetically silenced and does not produce mRNA [148]. The methylation pattern of the HPV genome varies with the viral lifecycle and with the degree of tissue lesions [147], suggesting that *de novo* methylation of HPV is a mechanism by which the host cell is activated to silence viral replication and transcription and even maintain long-term infection [149].

### 6.3 Molecular and functional effects of integration

Through massive sequencing analysis, different patterns of insertion of the viral genome into the host cell genome have been observed, the most frequent being the insertion of unaligned nucleotides (67 %), representing 27 % of the cases associated with integration, and the least frequent being the direct insertion of the entire HPV genome, representing only 6 % of cases [128]. Chromosomes with more than two integration sites that have been described to date include chromosomes 1, 2, 3, 4, 5, 6, 7, 8, 9, 10, 11, 12, 13, 14, 15, 17, 18, 19, 20 and X. Interestingly, chromosome 21 has not yet been reported as being susceptible to virus integration [125]. These chromosomes harbor genes associated with cancer development and progression, such as MYC, KLF5, KLF12, FHIT, HIF1A, RB1, TP63, TP53, BRCA2, LPR1B, PIK3CA, RBL1, PTEN, AKT1, TERT, CCND1, RICTOR, NOTCH1, KRAS and MTOR. Viral genome integration at these sites promotes the amplification or silencing of genes in different types of HR-HPV-associated cancers [137,[150], [151], [152], [153], [154]]. Enrichment pathway analysis revealed that these genes are associated with signaling pathways such as PI3K, WNT-catenin, and P53, among others, and that they have a direct impact on the main hallmarks of cancer, such as apoptosis inhibition, immortalization, evasion of the immune response, metabolic reprogramming, metastasis, and angiogenesis (Fig. 3).

HPV integration does not always result in increased E6/E7 expression [155]; it has been observed that, in different types of cancer, the integration of HPV 16 results in the amplification of transcription factors, protooncogenes, and tumor suppressors associated with cell proliferation and tumor progression due to the effect of the insertion of the viral genome in different regions of the genome, such as promoter regions (Table 2) [13].

#### 6.3.1 Cervical cancer

Analysis of next-generation sequencing data (RNA-seq and DNA-seq) has revealed HPV genome integration sites in premalignant lesions and cervical carcinoma. In cervical premalignant and malignant lesions, Hu et al. (2015) showed that HR-HPV integration inhibits or activates genes that favor the clonal selection of cells and, consequently, progression to cancer. Specifically, these researchers demonstrated that HR-HPVs can integrate into intergenic regions of tumor suppressor genes (FHIT and LRP1B POU5F1B) in regions bordering protooncogenes (MYC, HMGA2, KLF5, KLF12, FHIT, DLG2, and SEMA3B).

Holmes et al. (2016) reported that integration occurs most frequently in intergenic regions of genes associated with tumor progression (KLF5, KLF12, and MYC) in addition to those involved in the regulation of cell proliferation (RB1, AKT3, SST, and ID1); cell adhesion and motility (LPP); transcription factors such as AFF3, BCL6, CCAT1, and CCAT2; protooncogenes such as RAB11A and RAB22A; regulators of microtubule polymerization (MAST4 and MAP2); components of the extracellular matrix (MMP12 and COL4A4); and genes involved in angiogenesis (PF4V1) [156].

Few studies have focused on the analysis of HR-HPV genome integration in premalignant lesions and cervical cancer. Garza-Rodriguez et al. (2021) reported that type 2 integration is the most frequent type of integration in individuals with in multiple infections (HPV 51, 52, 45, and 31), and this type of integration occurs in tumor suppressor genes (RAD51B, MACROD2, FHIT, CSMD1, LRP1B, and DLG2) as well as genes involved in differentiation (CSMD3), cell migration (ROBO2), and histidine methylation (SETD3). These researchers noted that when viral genome integration occurs in intronic regions, there is a loss of protein function [157].

Yang et al. (2020) reported that integration occurred in intergenic regions on chromosome 20 (genes CHMP4B, RALY-AS1, KIF3B, and ASXL1), the X chromosome (genes RPS6KA3 and CNKSR2) and chromosome 6 (in the intronic region of the CAGE1 gene), and the genes located in these regions are considered protooncogenes and are involved in proliferation, migration, invasion and metastasis [117].

#### 6.3.2 Anal cancer

Although HR-HPV-positive squamous cell anal carcinoma patients have fewer genetic alterations and better responses to treatment than HPV-negative squamous anal cell carcinoma patients do [158], it is essential to consider alterations caused by viral integration in the search for therapeutic targets and to improve the clinical diagnosis and response to treatment.

Chung et al. (2016) analyzed anal squamous cell carcinomas positive for HPV 16 and 18 and reported alterations in genes involved in DNA repair, chromatin remodeling, and receptor tyrosine kinase signaling, as well as modifications in the copy numbers of genes involved in stem cell self-renewal and maintenance, cell growth, survival, proliferation, lethality and angiogenesis (SOX2, MYC, RICTOR, and PIK3CA) [159].

Using whole exome sequencing (WES) in primary and recurrent HR-HPV-positive squamous anal cell carcinoma tumors, Mouw et al. (2017) reported four recurrent mutations in the FBXW7 gene, an E3 ubiquitin ligase that targets protooncogenes such as c-MYC and cyclin E; mutations in PIK3CA; alterations in the KEAP-1 binding domain; and mutations in TP63 and EP300, both of which are involved in squamous cell differentiation [160].

Aldersley et al. (2021) analyzed anal squamous cell carcinomas associated with HPV 16 and 18 infections and reported that viral genome integration is associated with the silencing of BRCA2; the DNA damage response genes FOXO1, RB1, ATR, FANCD2, FHIT, MLH1, SETD2 APC, MSH3, PARP3, RAD18, RAD50, and XPC; and the amplification of protooncogenes (CCND1, MYC, NOTCH1, TERT, and PIK3CA) [142].

#### 6.3.3 Head and neck cancer

However, the role of HR-HPV infection in head and neck cancer is debatable given the strong effects of behavioral factors such as smoking and alcoholism in this type of cancer. HR-HPV-associated cancers have increased in incidence in young populations, and the role of viral genome integration has begun to make sense in the analysis of the progression of premalignant lesions to cancer.

Parfenov et al. (2014) analyzed HR-HPV integration in head and neck cancer and reported that it occurs in genes involved in cancer development, such as the protooncogenes RAD51B, ETS2, NR4A2, TPRG1, TP63, and KLF5 and in the intron of CD247 (programmed death ligand 1 PDL1), demonstrating that the integration of HR-HPV is associated with somatic alterations of critical genes involved in cancer development [13].

Walline et al. (2017) reported that viral genome integration occurs in the intronic regions of tumor suppressor genes (PTPRN2, ATR, TP63, and DCC) as well as in the intergenic regions, exons, and promoter regions of genes involved in cell differentiation processes and the regulation of gene expression (ETV6, TEMEM237, PGR, and TERT) [143]. Finally, Pinatti et al. (2021) identified eight integration sites in the intergenic region of genes associated with actin dynamics, migration, and metastasis and transcription factors involved in the regulation of microtubule dynamics, synaptic function, and neuronal morphology in HPV 16- and 18-positive oropharyngeal cancer [161].

#### 6.3.4 Penile cancer

Penile cancer is associated with pathologies such as lichen sclerosus, which is not associated with HPV infection. However, a higher incidence associated with HR-HPV infection and viral genome integration has been reported in young men. Therefore, studies based on next-generation sequencing have been conducted to identify new therapeutic targets to improve early diagnosis [162].

Annunziata et al. (2012) reported that HPV 16 integration in penile cancer occurs at chromosomal locus 8q21.3 of the FAM92A1 gene and locus 16p13.3 of the TRAP1 gene in intronic regions. TRAP 1 is a chaperone involved in cell differentiation, protein folding, and degradation, whereas FAM92A1 acts as a positive regulator of Hedgehog signaling [63]. Busso-Lopes et al. (2015) detected 28 copy number alterations in 20 % of HPV 16- and 18-positive penile carcinoma cases with viral integration, and gene amplification was observed on chromosomes 3q, 5p, 8q, 9p, 21p, and Y genes. The most frequent alterations were found on chromosomes Y, 3, and 8 [163]. Huang et al. (2021) analyzed the integration of HPV 16, 51, 33, and 56 in squamous cell penile cancer, identifying 2252 integration sites, including intragenic and intergenic regions of genes encoding transcription factors that regulate the expression of genes related to stemness, proliferation, apoptosis, autophagy and migration (KLF5, LRP1B, and KLF12) [153].

#### 6.3.5 Vulvar cancer

Although the etiology of vulvar cancer is associated with noninfectious processes, such as chronic dermatitis in postmenopausal women, this type of virus-free cancer generates more mutations compared with mutations in vulvar carcinoma cases caused HR-HPV infection and integration [164]. Moreover, the role of viral integration in tumor development and progression has also been evaluated [165].

Weberpals et al. (2017) reported that the mutation rate in HPV-negative vulvar carcinoma cases was 90 %, and mutations occurred in genes involved in DNA repair, migration, invasion, and cell cycle regulation (TP53, HRAS, PIK3CA, and CDK2A). In HPV 16-, 18-, 31-, and 33-positive vulvar carcinoma cases, a mutation rate of 73 % was observed, and mutations mainly occurred in genes involved in proliferation, differentiation, and apoptosis (PIK3CA, FGFR3, and PTEN) [166].

Han et al. (2018) identified nonsense mutations in HPV 16-, 58-, and 53-positive squamous vulvar cell carcinoma samples in previously described genes involved in growth, survival, proliferation, motility, cell morphology, DNA repair, and protein degradation (PIK3CA, BRCA2, and FBXW7) [167]. Mutations were also reported in previously undescribed genes involved in the organization of the extracellular matrix cytoskeleton (ERC1), regulators of T-cell differentiation and survival (BCL11), a key regulator of transcription elongation and regulator of gene expression involved in DNA repair (CDK12), a coactivator of nuclear receptors and stimulates transcriptional activity in a hormone-dependent manner (NCOA1) and a gene that induces the growth and differentiation of epithelial, glial, neuronal and skeletal muscle cells (NRG1) [168,169]. On the other hand, these researchers reported that tumor suppressor genes are a product of viral integration and that these genes are involved in cell cycle regulation, apoptosis, necrosis activation, transcriptional regulator (TP53), cell polarization, directed cell migration and modulation of cell-cell contact (FAT1), double-strand break repair and homologous recombination (BRCA2), molecular switch for apoptosis, necroptosis and pyroptosis necessary to prevent tissue damage (CASP8), and inhibition of metastasis and the epithelial‒mesenchymal transition (SMAD2) [167].

Williams et al. (2020) analyzed a group of vulvar squamous cell carcinoma cases with and without HR-HPV infection and reported more frequent point mutations in the PIK3CA, PTEN, EP300, STK11, AR, and FBXW7 genes. However, they reported more alterations in cancer samples without viral infection in TP53, TERT, CDKN2A, CCND1, FAT1, and NOTCH1, which are associated with proliferation, cell cycle invasion, and differentiation. EGFR and PDL1/PDL2 amplification was also observed [154].

HPV integration into the host genome is unnecessary for the viral life cycle and may not be required for cellular transformation. However, HPV integration occurs in genetic regions involved in cancer development and impacts key carcinogenic molecules, such as protooncogenes, tumor suppressor genes, signaling regulators, proliferation, apoptosis, and cellular repair mechanisms [170]. Data concerning the type of integration, the chromosomes that are susceptible to integration, and the genes that are affected by integration indicate that these events are related to the hallmarks of cancer, including altered proliferation (immortalization), genomic instability, resistance to cell death, activation and evasion of the immune response, dysregulation of cellular energetics (metabolic plasticity) and metastasis (Fig. 4). On the basis of the evidence presented in this review, it could be suggested that the concept of random integration should be reanalyzed in the context of HR-HPV infection. The effects of integration in CIN 1 should be seriously considered, as it is directly related to the progression of premalignant lesions to cancer.

## 7 Regression, persistence, and progression

Papillomaviruses are highly contagious, and approximately 40–60 % of people are infected in their first unprotected sexual intercourse [171], which is associated with the likelihood of generating a premalignant lesion. In addition, various factors such as coinfection or multiple infections with more than two HR-HPV genotypes (100) [172], viral persistence (the detection of the same HPV genotype in two or more screening tests within less than one year in an infected patient) [173], viral load [174], viral genome integration [157], and host factors, such as multiple sexual partners [175], smoking [176] and constant estrogenic stimulation [177], among others, can promote the progression from an early lesion to cancer [178].

Although HPV 16 and 18 are the most frequent genotypes, an increase in the incidence of HPV 33, 45 [179], 58 [180], 53 and 52 [181] has been associated with viral infection persistence and the progression of CIN 1 in young women. Multiple infections may increase the persistence rate of viral infection, thus impacting the oncogenic potential of the virus and the subsequent development and progression of premalignant lesions [182].

The immune system usually clears HR-HPV infections [183]. However, the persistence of infection in a small percentage of patients [184] is typically the starting point for various molecular mechanisms involving HPV type (single or multi-infection), viral load, and the physical state of the virus (episomal, integrated, or mixed) to converge and promote cancer development [185].

## 8 Conclusion

In HR-HPV-related infections, the ability of viral genome integration into the host cell genome to induce the overexpression of the E6 and E7 oncoproteins needs to be reconsidered [85]. Integration is thought to be a random process in early lesions. The most common is type 2 integration, where viral genome disruption initially occurs at sites such as E1, E2, E5, L1, L2, and LCR and then insertion into the host cell genome occurs through microhomology at regions susceptible to replication stress as well as transcriptionally active and DNase-susceptible regions, such as intergenic or intragenic regions. to After integration, the amplification of protooncogenes such as MYC, RAD51C, and PI3KA and the silencing of tumor suppressor genes such as p53 and PTEN result in alterations in cellular processes related to hallmarks of cancer. HR-HPV genome integration generates cellular phenotypes that impact tumor cell transformation at multiple sites in the human genome where the virus integrates, and the functional effects generated during the initiation of a premalignant lesion that could progress to cancer and the anatomical sites highlighted in this review must be considered. Integration is a determining factor in generating phenotypes associated with the progression of premalignant lesions to cancer in the different tissues infected by HR-HPVs. The assessment of integration and the molecules that are altered should be evaluated in early lesions and infected tissues without apparent morphological alterations.

## Funding statement

Catalán-Castorena was partially funded by CONAHCyT (470774).

## Data availability statement

No data was used for the research described in the article.

## CRediT authorship contribution statement

**Oscar Catalán-Castorena:** Writing – review & editing, Writing – original draft, Methodology, Conceptualization. **Olga Lilia Garibay-Cerdenares:** Writing – review & editing, Writing – original draft, Visualization, Supervision, Investigation, Formal analysis, Conceptualization. **Berenice Illades-Aguiar:** Writing – review & editing, Supervision, Investigation, Conceptualization. **Hugo Alberto Rodríguez-Ruiz:** Writing – review & editing, Methodology, Investigation, Conceptualization. **Ma. Isabel Zubillaga-Guerrero:** Writing – review & editing, Methodology, Investigation, Conceptualization. **Marco Antonio Leyva-Vázquez:** Writing – review & editing, Supervision, Investigation, Conceptualization. **Sergio Encarnación-Guevara:** Writing – review & editing, Supervision, Investigation, Conceptualization. **Luz del Carmen Alarcón-Romero:** Writing – review & editing, Writing – original draft, Visualization, Supervision, Methodology, Funding acquisition, Formal analysis, Conceptualization.

## Declaration of competing interest

The authors declare that they have no known competing financial interests or personal relationships that could have appeared to influence the work reported in this paper.
